# Correlations between TSC Expression and Menorrhagia in Adenomyosis Patients

**DOI:** 10.1007/s43032-026-02141-4

**Published:** 2026-07-21

**Authors:** Ni-Hao Gu, Liu-Jing Luo, Nai-Ping Yang, Ye-Ping Yang, Guo-Jing Li, Jing Ou-Yang, Chen-Xuan Wei, Yu Lin, Feng Sun, Si-Qin Yang, Hong Xu

**Affiliations:** 1 International Peace Maternity and Child Health Hospital, School of Medicine, Shanghai Jiao Tong University, Shanghai, 201403 China; 2 Shanghai Key Laboratory of Embryo Original Diseases, International Peace Maternity and Child Health Hospital, School of Medicine, Shanghai Jiao Tong University, Shanghai, 201403 China; 3 Shanghai Municipal Key Clinical Specialty, International Peace Maternity and Child Health Hospital, School of Medicine, Shanghai Jiao Tong University, Shanghai, 201403 China

**Keywords:** Adenomyosis, TSC expression, Menorrhagia, VEGFs

## Abstract

The TSC complex, formed by the binding of TSC2 with TSC1 and TBC1D7, plays an important role in the onset of endometrial-related diseases through the TSC-mTORC1 axis. However, the relationship between these proteins and adenomyosis has not been determined thus far. Here, we aimed to investigate the expression of TSC proteins in adenomyosis and determine the correlation between TSC expression and clinicopathologic parameters in patients with adenomyosis. 21 patients (age range, 40–50 years) with histologically diagnosed adenomyosis who underwent hysterectomy for nonendometrial disease were enrolled in this study. Specimens of healthy endometria were obtained from 21 patients (age range, 38–53 years) with cervical carcinoma in situ who underwent laparoscopy. The participants were interviewed via a standard questionnaire consisting of items pertaining to sociodemographic characteristics and reproductive history. The severity of dysmenorrhea and menorrhagia was quantified by means of the visual analogue scale and the menstrual pictogram, and preoperative hemoglobin levels were determined. Samples of serum and endometrial tissue were collected, TSC and VEGF expression was determined via immunofluorescence, and VEGF expression in the serum was quantified via ELISA. We found that, in patients with adenomyosis, TSC expression was significantly decreased during the secretory phase. Endometrial TSC1 and TSC2 expression was correlated with menstrual volume. Additionally, high levels of VEGFD increased the likelihood of moderate-to-severe menorrhagia in adenomyosis patients. Our results suggest that TSC is associated with clinical symptoms such as menorrhagia. In addition, VEGFD may be a potential quantitative predictor of the severity of menorrhagia in patients with adenomyosis.

**Supplementary Information:**

The online version contains supplementary material available at 10.1007/s43032-026-02141-4.

## Background

Adenomyosis (AM) is a benign uterine disease characterized by the presence of an ectopic endometrium and adjacent smooth muscle hyperplasia in the uterine muscle layer [1] and is considered one of the main causes of pelvic pain, abnormal uterine bleeding, and infertility [2]. According to current research findings, AM can result from epithelial–mesenchymal transition, ovarian steroid hormone dependence or resistance, abnormal immune function, oxidative stress, and free radical metabolism [3], suggesting the role of the endometrium, the mechanism of tissue injury and repair (TIAR), and the stem cell theory. However, its precise aetiology and physiopathology remain unknown [4–6]. Currently, considerable evidence suggests that the TSC–mTORC1 axis plays a principal role in the onset of endometrial-related diseases.

The TSC complex, formed by the binding of Tuberin (TSC2 protein) with Hamartin (TSC1 protein) and TBC1D7, is located at the centre of several important signalling pathways and regulates downstream molecules through posttranslational modifications and other means. A previous study revealed that TSC1 and TSC2 play a role in the insulin/phosphocreatine 3-kinase (PI3K)/Akt pathway and demonstrated that Akt regulates the TSC complex by directly phosphorylating TSC2 [7]. Usually, TSC complex can integrate stimuli from various extracellular factors, negatively regulate the activity of mTORC1 complexes, and play a role in tumour suppression [8]. The TSC2 protein (Tuberin) is considered a tumour suppressor that can stimulate specific GTPases, through which the C-terminal domain of TSC2 can inhibit the activity of the small GTPase Rheb, leading to Rheb-dependent inhibition of mTORC1 and activating its downstream effector molecules S6 kinase 1 and eukaryotic initiation factor 4E binding protein 1, ultimately inhibiting and regulating cell growth and proliferation [9]. Takiko Daikoku et al. reported that heightened mammalian target of rapamycin complex 1 (mTORC1) activity by genetic deletion of its direct inhibitor, TSC1, is associated with aberrant development and dysfunction of the female reproductive tract in mice [10]. Mutations in the tuberous sclerosis (TSC) gene can lead to constitutive activation of targets in the rapamycin (mTOR) pathway, causing dysregulation of cell proliferation, such as tuberous sclerosis and lymphangioleiomyomatosis [11]. In their study of endometrial cancer cell lines, Lu et al. reported that the activation of mTORC1 signalling is a common characteristic of individual primary endometrial cancer occurrence and that the loss of TSC2 protein expression caused by AKT-mediated phosphorylation of the TSC2 protein at serine 939 is an important factor leading to tumour occurrence and is positively correlated with the high incidence of mTORC1 activation in endometrial cancer [12].

Our previous studies revealed that downregulation of TSC2 inhibits autophagy induction by overactivating the mTORC1 signalling pathway in endometrial cells, leading to excessive migration and epithelial‒mesenchymal transition (EMT) [13]. Moreover, TSC2 overexpression induces the opposite effect, and cell proliferation and migration returns to normal levels after mTORC1 antagonist treatment. Therefore, we speculate that hypoexpression of TSC2 in the endometrium may promote AM. In addition, James B Brugarolas et al. reported that TSC2 can regulate vascular endothelial growth factor (VEGF) through both mTOR-dependent and mTOR-independent pathways [14]. Furthermore, studies using both animal models of AM [15] and human AM lesion tissues [16] indicate the importance of angiogenesis in disease development. As such, we suppose that VEGF, which may be inhibited by TSC, also participates in the pathogenesis of AM.

On the basis of the pathological results, patients in the secretory phase were uniformly selected. Twenty-one patients (aged 40–50 years) with histologically diagnosed AM who underwent hysterectomy for nonendometrial disease were enrolled in this study. Healthy endometrial samples were obtained from 21 patients (age range, 38–53 years) with cervical carcinoma in situ who underwent laparoscopy. The participants were interviewed via a standard questionnaire consisting of sociodemographic characteristics and reproductive history. The severity of dysmenorrhea and menorrhagia was evaluated via the visual analogue scale (VAS), the menstrual cycle and preoperative hemoglobin. Samples of serum and endometrial tissue were collected, TSC expression was determined via immunofluorescence, and VEGF expression in the serum was determined via ELISA to further explore the correlation between TSC expression and clinical pathological parameters in AM patients.

## Materials & Methods

### Ethical Approval

A hospital-based case–control study was conducted at International Peace Maternity and Child Health Hospital affiliated with Shanghai Jiao Tong University School of Medicine. Ethical approval for sample collection was obtained from the Ethics Committee of the International Peace Maternity and Child Health Hospital affiliated with Shanghai Jiao Tong University School of Medicine (Approval No. GKLW2020-23). Informed consent was obtained from all individual participants included in the study.

### Tissue Collection

In this study, ectopic and eutopic endometrial tissues were collected from 21 patients (aged 40–50 years) who underwent hysterectomy solely because of progressive dysmenorrhea (*n =* 10), excessive menstruation (*n =* 2), or both (*n =* 9). Healthy endometrial samples were obtained from 21 in situ cervical cancer patients who underwent laparoscopic examination (aged 38–53 years). The exclusion criteria for the participants were endometrial abnormalities, pelvic endometriosis, fibroids, ovarian cysts, lesions, other obvious internal or surgical comorbidities, and the use of any steroid hormone therapy in the last 3 months (Supplementary Fig. 1). Each tissue sample was fixed in 4% formalin.

### Menstrual Pictogram

We collected detailed menstrual histories of 42 patients and counted the number and penetration level of sanitary towels needed during one menstrual cycle [6]. Menstrual pictograms were used to estimate menstrual volume, which divided towels into three lengths: normal, long, and night [17, 18]. Each was further divided into five grades based on the dyed area of 10, 25, 50, 75, and 100 cm^2^. The 5 grades of normal towels corresponded.

### Immunofluorescence

For the immunofluorescence analysis, the paraffin-embedded sections were dewaxed and then subjected to heat-mediated antigen retrieval, which was performed by boiling the sections at high pressure for 20 min in antigen repair solution (G1202; Servicebio, Wuhan, China). The sections were allowed to cool and briefly rinsed three times in PBS, each time for 5 min. The sections were then incubated for 30 min in 10% donkey serum and again overnight at 4 °C in solution containing the relevant primary antibody, namely, TSC1 (D43E2; CST, MA, USA), TSC2 (D93F12; CST, MA, USA), VEGF-A (EP1176Y; Abcam, Cambridge, UK) or VEGFD (EPR8457; Abcam, Cambridge, UK) at the appropriate dilution. The corresponding secondary antibody was added, and the samples were incubated at room temperature in the dark for 50 min. Then, the cells were counterstained with DAPI for 10 min. All the sections were incubated under the same conditions with the same antibody concentration.

The tissue sections were observed and photographed under a microscope, and semi-quantification was performed via ImageJ software. The comprehensive optical density (IOD) of each image was calculated. Five regions from each slice were randomly selected for measurement, and the images were quantified via the immune response area (IA) (μm2) and IOD. Software was used to circle the glandular cells and collect the comprehensive optical density of the glandular cells (circled part) and the stromal cells (outside the circled part). Similarly, IA and IOD were used for quantification. Finally, the staining intensity (SI) of each image was calculated as SI = IOD/IA.

### ELISA

Before treatment, peripheral blood was collected from the patient between days 2 to 5 days of a spontaneous menstrual cycle, and an enzyme-linked immunosorbent assay was used to detect serum VEGF-A (sc-7269; SCBT, Texas, U.S.A.) and VEGFD (EHFIGF; Thermo, Massachusetts, U.S.A.) levels.

### Statistical Analysis

**Fig. 1:**
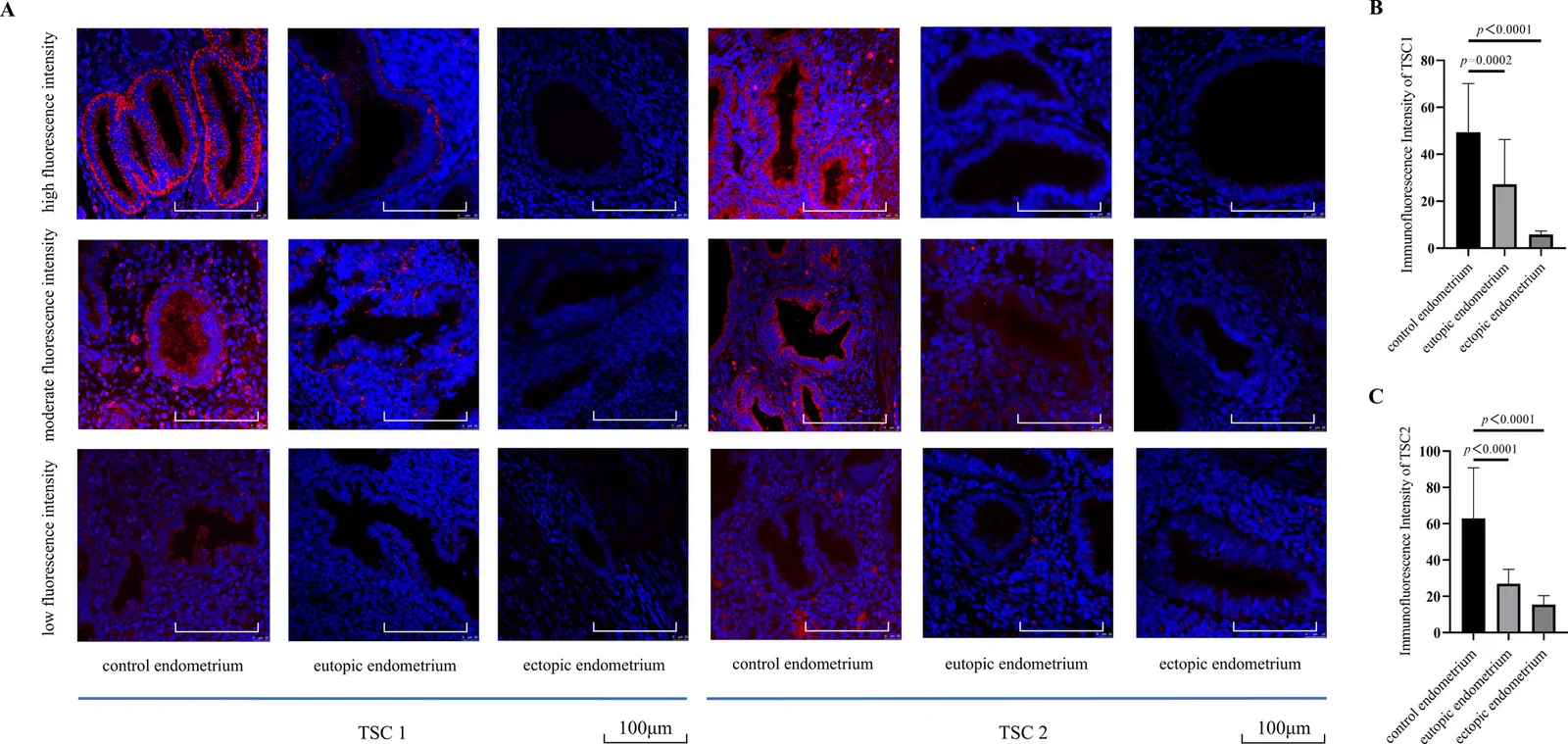
Expression of TSC in AM during the secretory phase. **A** Representative immunofluorescence images of TSC1 and TSC2 in the endometrial tissues of the control group and those in the endometrial tissues or lesions of patients with AM in the secretory phase. Scale bars, 100 µm. **B** The expression levels of TSC1 in the control endometrium were higher than those in the eutopic endometrium and ectopic lesions. **C** The expression levels of TSC2 in the control endometrium were higher than those in the eutopic endometrium and ectopic lesions

The data were presented as the means ± SD. The statistical results were analysed via GraphPad Prism. All comparisons were analysed with unpaired two-tailed t tests or correlation coefficient analysis followed by linear regression, except for the results in Fig. 1B, C, where were analysed with one-way *ANOVA*. Corrections for multiple comparisons were applied by Tukey's multiple comparisons test. Normality assumptions were tested. The data followed a normal distribution or an approximate normal distribution. No outliers or missing data involved.

## Results

### Patient Characteristics

Table 1 presents a summary of the demographic characteristics of the study subjects. A total of 42 patients were recruited, and all participants were of Chinese origin. Pairs of endometrium samples (i.e., eutopic endometrium and ectopic endometrium samples from the same patient) were collected from 21 patients with AM who underwent hysterectomy for progressive dysmenorrhea or menorrhagia. Healthy endometrial samples were obtained from 21 patients with cervical carcinoma in situ who underwent laparoscopy. In particular, all 42 patients included in this study were confirmed to be in the secretory phase by pathology. There were no significant differences in age, age of menarche, gravidity, or parity between the AM and control groups (*p* = 0.675; *p* = 0.449; *p* = 0.625; *p* = 0.172 respectively). Statistical analysis revealed that the menstrual volume, hemoglobin concentration, VAS score, and uterus size significantly differed between the AM and control groups (all *p* < 0.001). These findings are consistent with the clinicopathologic parameters observed in AM patients.

**Table 1:** Characteristics of recruited patients with AM and controls

	AM (*N =* 21)	Control (*N =* 21)	*p*-value
	*n* (%) or mean (SD)	*n* (%) or mean (SD)	
Age	43.43 (3.41)	43.95 (4.54)	0.675
Age of menarche	13.81 (1.12)	14.14 (1.65)	0.449
Menstrual day	6.24 (1.55)	4.98 (1.77)	0.019
Menstrual cycle	26.83 (2.86)	28.31 (4.76)	0.23
Menstrual volume	76.38 (26.76)	43.33 (16.86)	< 0.001
Hemoglobin concentration	100.76 (22.51)	126.14 (12.33)	< 0.001
VAS score	7.43 (2.09)	0.14 (0.66)	< 0.001
The volume of the uterus	299.20 (146.99)	61.39 (23.36)	< 0.001
The frequency of pregnancy			0.625
≤ 2	7 (33.3)	6 (28.6)	
3	7 (33.3)	10 (47.6)	
≥ 4	7 (33.3)	5 (23.8)	
The frequency of childbirth			0.172
≤ 1	17 (81.0)	13 (61.9)	
≥ 2	4 (19.0)	8 (38.9)	

The uterus size was calculated as (π * D1 * D2 * D3)/6, where D1 = the distance from the fundus to the internal os of the cervix, D2 = the transverse diameter at the level of the cornua, and D3 = the anteroposterior diameter at the level of the cornua

Data are presented as the number (percentage) or mean (SD)

*VAS* visual analogue scale, *SD* standard deviation

### Epithelial TSC Expression is Significantly Decreased during the Secretory Phase

42 subjects were recruited, including those with and without AM, and human endometrial biopsies were collected. TSC expression was then analysed by means of immunofluorescence (IF) staining, which consistently revealed that TSC was widely expressed in endometrial tissue, particularly in epithelial cells, and was localized in the cytoplasm (Fig. 1A). The expression of both TSC1 and TSC2 was significantly lower in both the epithelial and stromal cells of the AM eutopic endometria than in those of the control endometria (*p* < 0.0001, *p* = 0.0002; Fig. 1B, C). Furthermore, the expression of TSC1 and TSC2 was evaluated in AM tissues, including 21 pairs of ectopic and eutopic specimens, by means of IF staining. The expression of TSC1 and TSC2 in the lesions was also significantly lower than that in the control endometria (*p* < 0.0001, *p* < 0.0001; Fig. 1B, C). These findings validate the hypothesis that TSC is hypo-expressed in AM during the secretory phase. However, due to the low expression of TSC in lesions, considering accessibility and reliability, only the expression level of TSC in the eutopic endometrium of AM patients was used to explore the correlation with clinicopathologic parameters.

### Correlation between TSC Expression and Clinicopathologic Parameters in 42 Patients

**Fig. 2:**
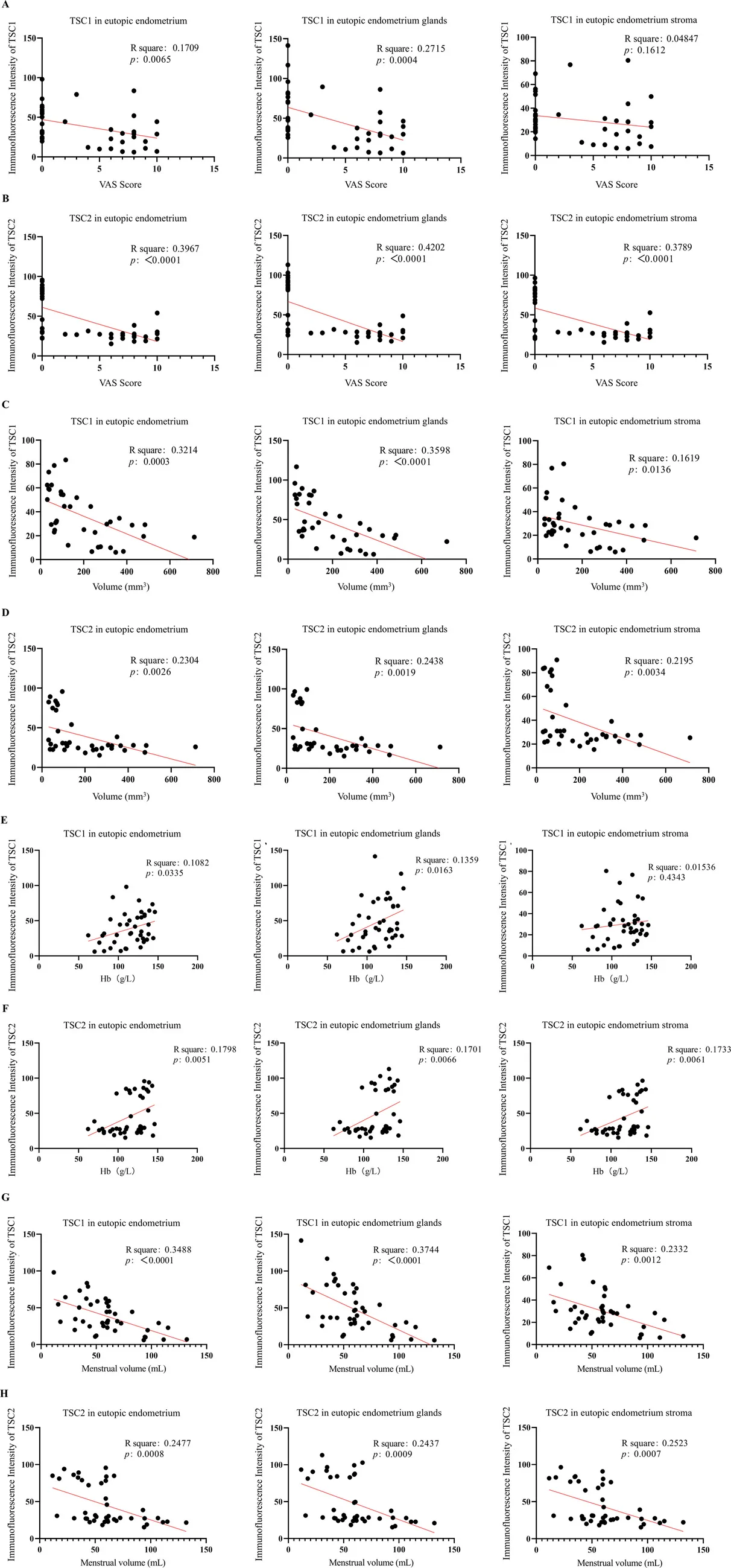
Correlation between TSC expression and clinicopathologic parameters in 42 patients. **A**, **B** The correlation between the expression levels of TSC1 and TSC2 in the eutopic endometrium and in the glands or stroma and the VAS score of all patients. **C**, **D** The correlation between the expression levels of TSC1 and TSC2 in the eutopic endometrium and in the glands or stroma and the uterus volume of all patients. **E**, **F** The correlation between the expression levels of TSC1 and TSC2 in the eutopic endometrium and in the glands or stroma and the minimum hemoglobin concentration three months prior to surgery of all patients. **G**, **H** The correlation between the expression levels of TSC1 and TSC2 in the eutopic endometrium or in glands or stroma and the menstrual volume of all patients

Correlation analysis revealed that TSC1 and TSC2 expression levels were negatively correlated with VAS scores (*p* = 0.0065, *p* < 0.0001 respectively; Fig. 2A, B) and uterine volume (*p* = 0.0003, *p* = 0.0026 respectively; Fig. 2C, D). In contrast, both TSC1 and TSC2 expression showed a positive correlation with the minimum hemoglobin level measured three months prior to surgery (*p* = 0.0335, *p* = 0.0051 respectively; Fig. 2E, F). Additionally, TSC1 and TSC2 expression were negatively correlated with menstrual volume (*p* < 0.0001, *p* = 0.0008 respectively; Fig. 2G, H). Concurrently, a correlation analysis was conducted between TSC expression and coagulation indices. No significant correlations were found between TSC1 and TSC2 expression and platelet count, prothrombin time, or other coagulation indices (Table 2). Overall, these may suggest a correlation between TSC expression and clinical symptoms, such as dysmenorrhea and menorrhagia.

**Table 2:** Correlation analysis between TSC expression and coagulation indices

		Platelet count	International normalized ratio	Actived partial thromboplastin time	Thrombin time	Prothrombin time	Concentration of Fibrinogen	D Dimer	Fibrin degradation products
TSC1 in endometrium	Correlation Coefficient	−0.1002	0.009506	−0.04895	0.1025	0.06079	0.01154	0.07725	−0.03277
	*p*	0.5278	0.9524	0.7582	0.5183	0.7021	0.9500	0.6356	0.8685
TSC1 in endometrium glands	Correlation Coefficient	−0.1323	0.03757	0.03813	0.1185	0.08428	0.03151	0.09940	−0.01367
	*p*	0.4037	0.8132	0.8105	0.4546	0.5957	0.8641	0.5417	0.9450
TSC1 in endometrium stroma	Correlation Coefficient	−0.01270	0.07657	−0.1347	0.004498	0.1168	0.03098	0.01285	−0.05717
	*p*	0.9364	0.6299	0.3950	0.9774	0.4615	0.8663	0.9373	0.7726
TSC2 in endometrium	Correlation Coefficient	−0.1098	0.2264	0.1133	0.09923	0.2222	0.1387	0.2328	0.1108
	*p*	0.4888	0.1493	0.4751	0.5318	0.1572	0.4492	0.1483	0.5747
TSC2 in endometrium glands	Correlation Coefficient	−0.1299	0.2859	0.1334	0.102	0.2758	0.1436	0.243	0.1334
	*p*	0.4122	0.0665	0.3995	0.5203	0.077	0.4331	0.1309	0.4984
TSC2 in endometrium stroma	Correlation Coefficient	−0.1009	0.2076	0.09987	0.1051	0.205	0.1392	0.2206	0.0932
	*p*	0.5247	0.1871	0.5292	0.5078	0.1927	0.4475	0.1714	0.6371

*p* represents the p value

### Correlation between TSC Expression and Clinicopathologic Parameters in 21 Patients with AM

To investigate whether the expression of TSC is correlated with the severity of AM, the correlation between TSC expression and clinicopathologic parameters in patients with AM was analysed. Unfortunately, there was no correlation between TSC1 and TSC2 expression and VAS score, the uterus volume or the minimum hemoglobin level at three months prior to surgery in the AM group (Table 3). However, TSC1, but not TSC2, was negatively correlated with the menstrual volume (Table 3). Therefore, we supposed that the level of TSC1 could be used to infer whether patients with AM experience or will experience menorrhagia.

**Table 3:** Correlation between TSC expression and clinicopathologic parameters in 21 patients with adenomyosis

	Menstrual volume		Haemoglobin level		VAS score		The volume of uterus	
	Correlation coefficient	*p*	Correlation coefficient	*p*	Correlation coefficient	*p*	Correlation coefficient	*p*
TSC1 in endometrium	−0.4709	0.0312	0.07659	0.7414	0.08919	0.7007	−0.4004	0.0721
TSC1 in endometrium glands	−0.4642	0.034	0.0491	0.8326	0.02256	0.9227	−0.3499	0.12
TSC1 in endometrium stroma	−0.4463	0.0426	−0.01249	0.9572	0.1101	0.6346	−0.359	0.11
TSC2 in endometrium	−0.2407	0.2932	0.1316	0.5696	0.1633	0.4794	−0.2248	0.3273
TSC2 in endometrium glands	−0.3022	0.183	0.08724	0.7069	0.1225	0.5967	−0.2285	0.319
TSC2 in endometrium stroma	−0.2123	0.3555	0.1225	0.5967	0.1553	0.5014	−0.2199	0.3381

*p* represents the p value

Red represent *p* ≤ 0.05

### Correlation between Serum and Endometrial Levels of Vascular Endothelial Growth Factor (VEGF) and the Severity of Menorrhagia in AM Patients

**Fig. 3:**
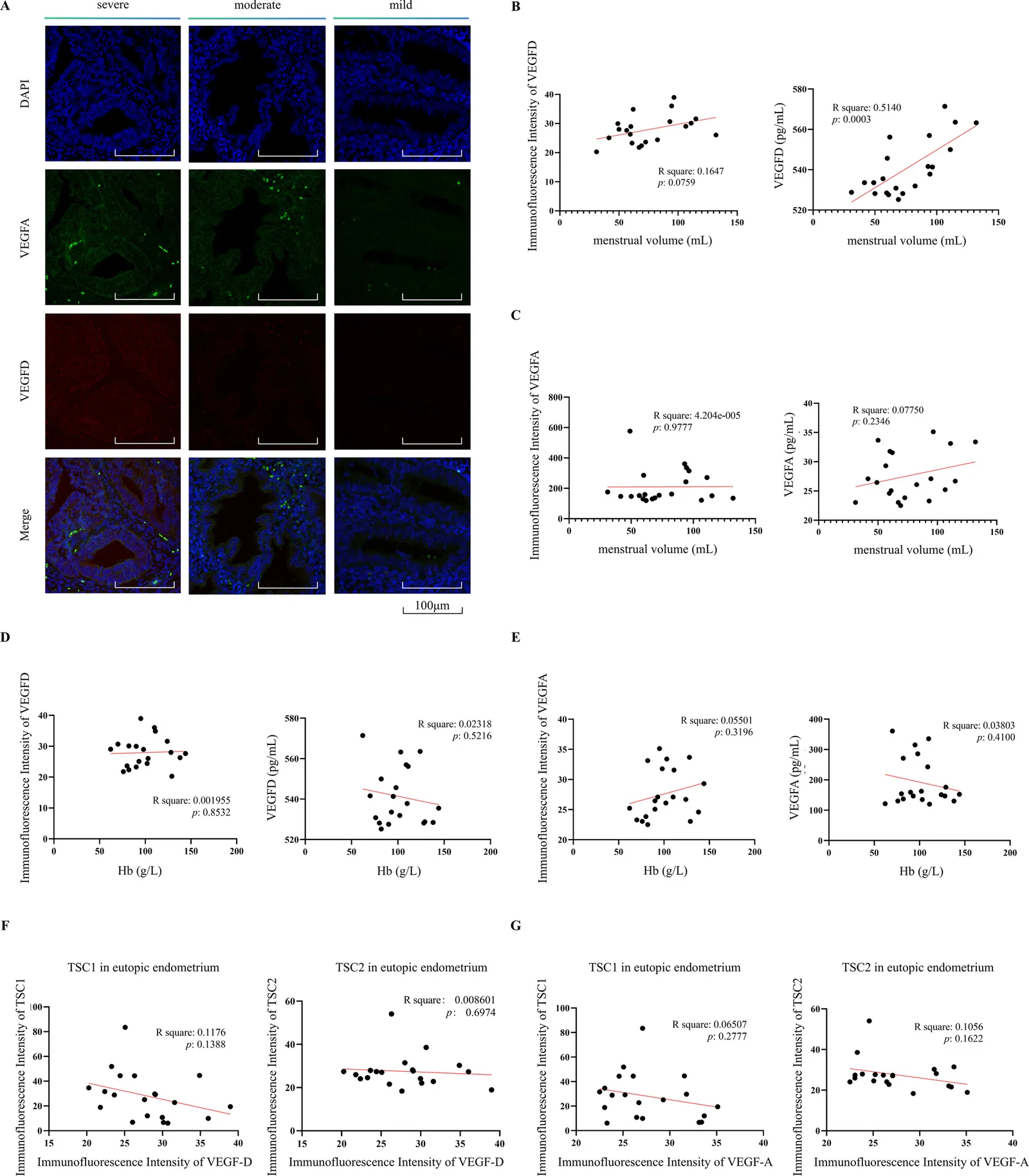
Correlations between the serum and endometrial levels of VEGFs and the severity of menorrhagia in AM patients. **A** Representative immunofluorescence images of VEGF in the endometrial tissues of patients with AM, divided into mild, moderate, and severe according to fluorescence intensity. Scale bars, 100 µm. **B**, **C** The correlation between the expression levels of VEGFD or VEGF-A in endothelial tissues or serum and the menstrual volume of AM patients. **D**, **E** The correlation between the expression levels of VEGFD or VEGF-A in endothelial tissues or serum and the minimum hemoglobin concentration three months prior to surgery of AM patients. **F**, **G** The correlation between the expression levels of TSC1 and TSC2 in the eutopic endometrium and the expression levels of VEGFD or VEGF-A in the endometrial tissues of AM patients

Finally, we examined the expression and content of VEGFs downstream of TSC in the endometrial and paired serum samples of patients with AM. The images presented correspond to specimens with different intensities of VEGFA and VEGFD staining, as determined by immunofluorescence (IF) staining (Fig. 3A). The results revealed a positive correlation between VEGFD expression in serum and the menstrual volume (*p* = 0.0003; Fig. 3B), whereas no such correlation was observed for VEGFA (Fig. 3C). Moreover, no correlation was identified between preoperative hemoglobin levels and either VEGFA or VEGFD expression in endothelial tissues or serum levels (Fig. 3D, E). Furthermore, we explored the relationship between TSC and VEGF in tissues and found no significant correlations (Fig. 3F, G).

**Fig. 4:**
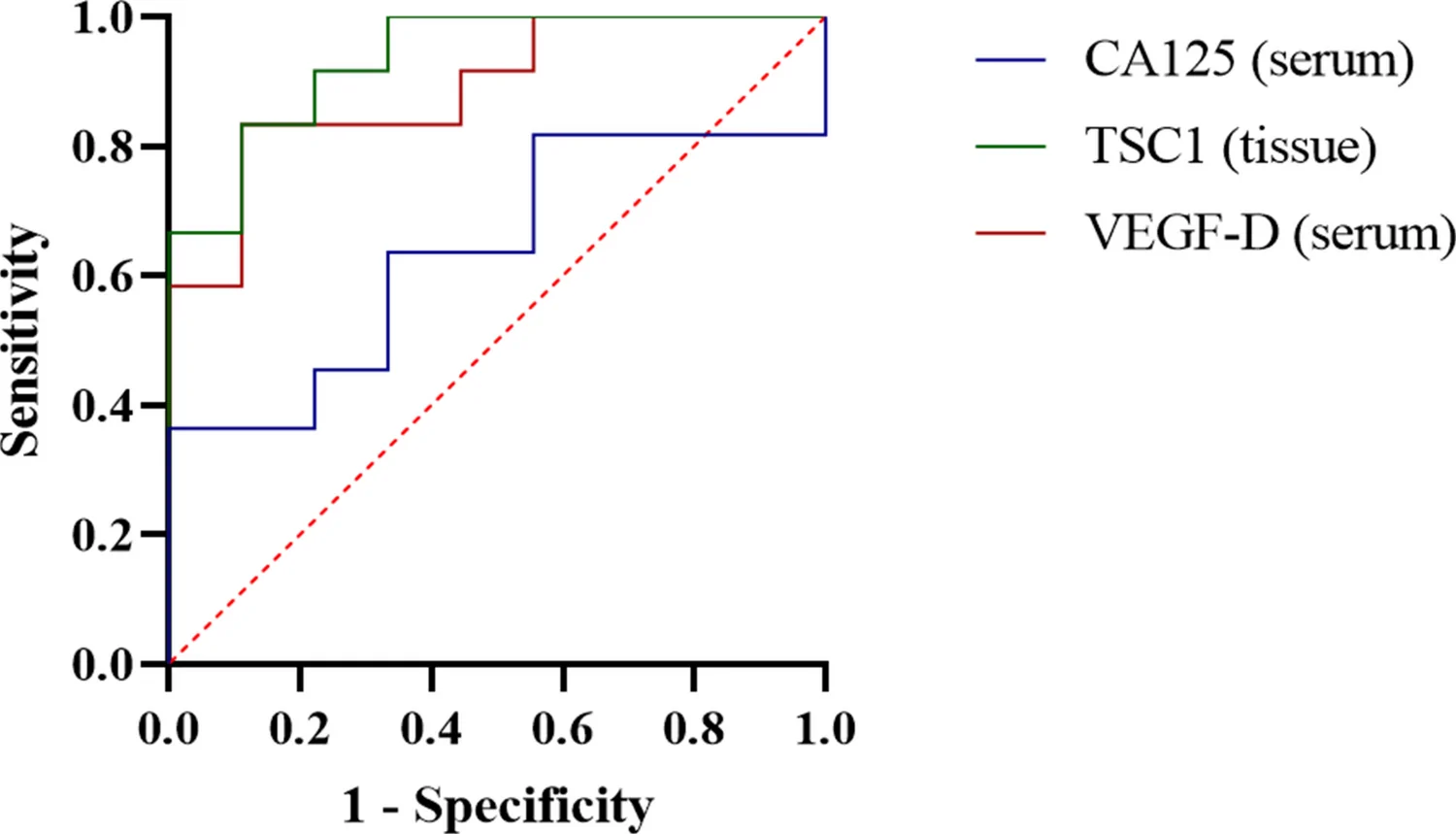
Receiver-operating characteristic (ROC) analysis of TSC1, VEGFD, and CA-125. Receiver-operating characteristic (ROC) analysis was used to assess the discriminatory performance of three biomarkers: endometrial TSC1 protein expression, serum VEGFD, and serum CA-125

In the 21 women diagnosed with adenomyosis (AM), menstrual blood loss exceeding 80 mL was classified as menorrhagia [19], whereas blood loss of ≤ 80 mL was considered normal. Receiver-operating characteristic (ROC) analysis was used to assess the discriminatory performance of three biomarkers: endometrial TSC1 protein expression, serum VEGFD, and serum CA-125 (Fig. 4). The areas under the ROC curve (AUC) were 0.935 (95% CI: 0.835–1.000; *p* = 0.0008) for TSC1, 0.889 (95% CI: 0.749–1.000; *p* = 0.0028) for VEGFD, and 0.636 (95% CI: 0.385–0.887; *p* = 0.305) for CA-125. Utilising the maximal Youden index (0.722), a cut-off value of 536.7 pg/mL was identified for VEGFD in the present dataset. However, the stability and generalisability of this specific threshold are uncertain and must be prospectively validated in an independent, larger patient population. Notwithstanding these limitations, the magnitude of the AUC and Youden index estimates suggests that TSC1 and VEGFD are promising biomarkers worthy of further investigation for distinguishing menorrhagia in AM patients.

## Discussion

In this study, TSC was again demonstrated to be hypo-expressed in the secretory endometrium of patients with AM. Importantly, for the first time, we revealed a correlation between TSC and bleeding indicators. We found that TSC is negatively correlated with the VAS score and uterus volume. In addition, TSC is positively correlated with the minimum hemoglobin level and negatively correlated with the menstrual volume, indicating that TSC may be associated with clinical symptoms such as dysmenorrhea and menorrhagia. Moreover, TSC1 was negatively correlated with the menstrual volume in patients with AM. Overall, these findings may suggest a correlation between TSC expression and the severity of AM. Our previous research also found that downregulation of TSC2 inhibits autophagy induction and leads to excessive migration and EMT by over-activating the mTORC1 signalling pathway in endometrial cells, which may promote AM. These results support our current conclusions.

Due to the difficulty in obtaining the expression level of TSC in patients, we further attempted to find an accessible detection index. According to previous research, TSC can regulate VEGF through both mTOR-dependent and mTOR-independent pathways [14]. VEGF is an effective endothelial cell mitogen that can increase vascular permeability and is involved in inflammatory processes and several pathological processes associated with increased angiogenesis [20]. There is considerable evidence to suggest that, compared with the endometria of healthy women, the endometria of both normal and abnormal AM patients exhibit higher VEGF expression and increased endometrial angiogenesis, indicating that VEGF plays an important role in the development of AM [16, 21, 22]. This conclusion has also been confirmed in animal models of AM [15]. In addition, lymphangiomyomatosis (LAM) is a rare disease, with almost all cases occurring in females, mainly in women of childbearing age, and lesions often present in the pulmonary lymphatic endothelial cell network. Meanwhile, research confirmed that the onset of LAM is associated with TSC mutations. Research also found that many LAM patients have elevated serum levels of VEGFD, and a VEGFD level > 800 pg/ml is one of the important diagnostic criteria for LAM [23]. Our results revealed a positive correlation between the serum level of VEGFD and the severity of menorrhagia in AM patients. Additionally, high levels of VEGF increased the likelihood of moderate-to-severe menorrhagia in AM patients. Therefore, VEGFD may be a potential quantitative predictor of the severity of menorrhagia in AM patients. Recently, single-cell RNA sequencing results have shown that ANGPT, TIE1, VEGFR1, and VEGFR2 are upregulated in the lesion area of AM, where they promote migration and proliferation to form new vascular structures and differentiate into various endothelial subtypes [24]. This abnormal vascular permeability and angiogenesis may be one of the reasons for symptoms such as excessive menstruation [24–26]. Given that TSC negatively regulates the mTOR pathway—which plays a crucial role in endothelial cell proliferation, VEGF signaling, and angiogenesis—our findings suggest that TSC dysregulation may represent an upstream mechanism driving the aberrant angiogenic activity observed in AM lesions. These results all confirm our conclusions.

Several inherent limitations of this study merit attention. First, the TBC1D7 has been identified as a component of the TSC complex; however, the results of our study revealed that its expression levels were comparable in both groups, with no statistically significant differences observed. Previous studies suggested that loss-of-function mutations in TSC1 or TSC2, but not TBC1D7, give rise to tuberous sclerosis complex (TSC) [27, 28]. TSC1 and TSC2 are sufficient to drive the observed phenotype, while TBC1D7 is not a core component and only plays an auxiliary role in enhancing the stability of the TSC complex. Second, the number of cases we included was not sufficiently high, and most were cases of diffuse AM, mainly due to our strict screening criteria for the included cases. For example, there was a potential positive correlation between the expression level of VEGFD in the endometrium and menstrual volume, but unfortunately it was not statistically significant, which must be prospectively validated in an independent, larger patient population. Third, we acknowledged that even situ cervical cancer (control group) might introduce subtle, localized inflammatory cues. Furthermore, we plan to use a mouse model to validate the above results in vivo, making them more reliable and convincing. 

## Conclusions

In summary, we validated the correlation between TSC expression and clinicopathologic parameters in AM patients. TSC may be associated with clinical symptoms such as menorrhagia. In addition, VEGFD may be a potential quantitative predictor of the severity of menorrhagia in AM patients. Our findings help to elucidate the aetiology of AM and provide a bridge between the pathogenesis and clinical manifestations of the disease.

## Supplementary Information

Below is the link to the electronic supplementary material.

Supplementary file1 (TIF 1187 KB)

## Data Availability

All the data generated or analysed during this study are included in this published article (and its supplementary information files).

## Abbreviations

AM: Adenomyosis
EMT: Epithelial–mesenchymal transition
IA: Immune response area
IF: Immunofluorescence
IOD: Integrated optical density
MVD: Micro vessel density
PI3K: Insulin/phosphocreatine 3-kinase
SI: Staining intensity
TBC1D7: TBC1 domain family member 7
TIAR: Tissue injury and repair
TSC: Tuberous sclerosis complex
VAS: Visual analogue scale
VEGF: Vascular endothelial growth factor
